# The impact of drug palatability on prescribing and dispensing of antibiotic formulations for paediatric patients: a cross-sectional survey of general practitioners and pharmacists

**DOI:** 10.1093/fampra/cmad071

**Published:** 2023-07-06

**Authors:** Ayat Elgammal, Joseph Ryan, Colin Bradley, Abina Crean, Margaret Bermingham

**Affiliations:** Pharmaceutical Care Research Group, School of Pharmacy, University College Cork, Cork, Ireland; SSPC Pharmaceutical Research Centre, School of Pharmacy, University College Cork, Cork, Ireland; Department of General Practice, School of Medicine, University College Cork, Cork, Ireland; Department of General Practice, School of Medicine, University College Cork, Cork, Ireland; SSPC Pharmaceutical Research Centre, School of Pharmacy, University College Cork, Cork, Ireland; Pharmaceutical Care Research Group, School of Pharmacy, University College Cork, Cork, Ireland

**Keywords:** antibiotics, antimicrobial stewardship, caregivers, paediatrics, patient adherence, primary care

## Abstract

**Background:**

Palatability is a key element of paediatric acceptability for medicines. Many patient and drug factors are considered when choosing an antibiotic for a child. Pharmacists report that they receive questions about the palatability of oral liquid antibiotics for children. This study aimed to explore the experiences of GPs and pharmacists concerning palatability of oral liquid antibiotics for children.

**Methods:**

A questionnaire about the impact of palatability on the choice of antibiotic formulation for children was emailed to all community pharmacists in Ireland and to GPs and trainee GPs in the Cork region and posted on social media. Survey items were not compulsory; therefore, percentage responses were calculated based on the number of responses to that item. GP and pharmacist responses were analysed independently.

**Results:**

Responses were received from 244 participants (59 GPs, 185 pharmacists). Clinical guidelines and availability of supply were the most important factors considered when choosing an oral liquid antibiotic formulation for children by GP (79.7%) and pharmacist (66.5%) respondents respectively. Forty GP respondents (76.9%) reported ensuring adherence was the most common palatability-related reason leading to deviation from guidelines. Pharmacist respondents (52%) reported advising a parent/caregiver to manipulate the required antibiotic dose to improve acceptability. The least palatable oral liquid antibiotics reported were flucloxacillin (16% GPs, 18% pharmacists) and clarithromycin (17% of each profession).

**Conclusion:**

This study identified palatability issues associated with oral liquid antibiotics for children reported by GPs and pharmacists. Pharmaceutical approaches to adapting oral liquid antibiotic formulations must be developed to improve palatability and thus paediatric acceptability.

**Key messages**

- Drug palatability affects antibiotic prescribing and dispensing for children.
- Palatability issues result in general practitioners deviating from guidelines.
- Pharmacists report switching antibiotics due to parent/caregiver feedback.
- Amoxicillin and cefaclor were noted as the most palatable antibiotics.
- The least palatable antibiotics noted were flucloxacillin and clarithromycin.
- Pharmaceutical approaches to improve oral antibiotic palatability are needed.

## Introduction

Palatability is *the overall appreciation of a medicinal product in relation to its smell, taste, aftertaste and texture (i.e. feeling in the mouth)*.^1^ Antibiotics prescribed for the treatment of common paediatric illnesses are noted for their poor palatability.^2–6^ The palatability of these oral antibiotic products is a key consideration to ensure compliance, adherence, and effective treatment.^7^ Poor palatability, leading to incomplete antibiotic treatment, can result in disease recurrence and can lead to the use of broad-spectrum antibiotics that is discouraged because it may then cause antimicrobial resistance and rising rates of resistance in the general population.^8–11^

Antibiotics are commonly prescribed paediatric therapeutic agents and the majority of pre-school children are prescribed one or more medications each year, most frequently antibiotics.^12–14^ However, prescribing the most suitable antibiotic for children is a challenging process even with an obvious diagnosis.^15^ General practitioners’ (GPs) considerations when prescribing antibiotics for children in a community setting are multi-faceted.^11,16–18^ After clinical indication and compliance with prescribing guidelines, palatability is a secondary factor that can influence the prescribing of oral antibiotics for children. Poor palatability of antibiotic formulations was also noted during a study exploring how formulation factors are considered by Norwegian GPs when prescribing medicines for children. The GPs expressed their preference to prescribe first-choice antibiotics according to clinical guidelines. However, when patient adherence was undermined by antibiotic palatability, it presented them with ‘two suboptimal alternatives’; prescribing a second-choice antibiotic course that the child may adhere to, or the first-choice course that might not be completed.^15^ The antibiotic example highlighted in this study was Penicillin V (phenoxymethylpenicillin), which was the first-choice antibiotic for common respiratory infections. However, in the study, this agent was reported to have poor acceptability and to be challenging to administer to children.

Bergene et al. also explored the consideration of formulation factors by pharmacists dispensing medicines for children.^15^ Pharmacists reported it was common to have questions from parents related to the taste of medicines prescribed for children. They reported that sometimes they dispensed a different formulation of an antibiotic prescribed if a patient indicated an inability to administer a specific formulation to their child. However, the antibiotic type was only changed after consulting the prescriber. Both GPs and pharmacists engage with the challenges of the paediatric acceptability of oral liquid antibiotics. The use of medications in paediatric patients can be enhanced through an appropriate support from healthcare professionals. They can support parents/caregivers by counselling on the importance of the treatment, and how to administer the medication to the child.^19^ While GPs and pharmacists are not the medicine takers, both professions receive feedback, in particular from parents/caregivers, about the acceptability and palatability of oral liquid antibiotics.^15^ Pharmacists have a unique perspective on the texture and smell as they are responsible for reconstituting oral liquid antibiotics from the dry powder state.

GPs and pharmacists are aware of the challenges of administering unpalatable oral medicines for children.^19–23^ However, studies investigating the influence of palatability on their prescribing and dispensing practice are limited. Previous studies on this topic have focussed on specific medicines^15^ or on specific infections,^16,18^ have not included pharmacists^16,18^ and have not focussed in detail on palatability. The aim of this study was to explore GPs’ experiences of prescribing oral liquid antibiotics for children and pharmacists’ experiences of dispensing oral liquid antibiotics for children.

## Methods

### Ethical approval

Ethical approval was granted by the Social Research Ethics Committee of University College Cork, Log number 2021-138.

### Study design

A survey was developed to maximise the reach of the study and obtain the views of a large sample of GPs and pharmacists. The draft questions were developed with reference to the literature review specifically qualitative studies conducted by Bergene et al., Venables et al., and Bradshaw et al.^15,20,24^ and the professional experience of the research team members. The research team consisted of two GPs and three pharmacists. All questions used in the survey are novel questions developed for this study by the research team. The draft questionnaire was piloted electronically. It was shared by email with an independent convenience sample of four GPs and four pharmacists. The responses to the survey and qualitative feedback from this sample were used to refine the survey and finalise the questions. These responses were not included in the final analysis. The final questionnaire (Supplemental Material) was agreed by all authors. The survey was hosted in Google Forms and consisted of a participant information sheet, consent form, and questionnaire.

### Questionnaire composition

The questionnaire consisted of 20 questions for GP and 23 questions for pharmacist participants. The initial sections collected demographic data. The GP branch of questions focussed on factors that influence the prescribing of oral liquid antibiotics, whereas the pharmacist branch focussed on factors that influence the dispensing of oral liquid antibiotics. The final sections, for GPs and pharmacists, contained questions about the palatability of oral liquid antibiotics. To explore the antibiotics most associated with general palatability issues in children, all participants were asked to select from a given list of the oral liquid antibiotics. As pharmacists reconstitute oral liquid antibiotic products prior to dispensing they are exposed to their aroma. Some antibiotics have been reported to have a foul smell.^4,25,26^ To explore this further, pharmacists were asked to state oral liquid antibiotic products with smell issues that they had encountered during their preparation. Pharmacists were also asked to state the oral liquid antibiotic products with texture issues they had encountered during their preparation. Survey questions 11, 12, 15, and 16 used a four-item ranking scale from frequently to never. Questions 9 for GPs and question 10 for pharmacists ranked the factors considered when prescribing or dispensing an oral liquid antibiotic product for children. Respondents were asked to rank 1 for the factor considered most important, 2 for the next most important, and so on.

Most of the survey questions were not compulsory and respondents had the option to skip these questions. For some questions, one or more answers could be chosen by respondents. The only compulsory questions were questions 9, 12, 13, and 17–19 in the GP survey and questions 10, 13, and 15–17 in the pharmacist survey. The research team limited the number of compulsory questions to ensure that participants answered only the questions of which they had knowledge or experience. A child, for the purposes of this study, was considered to be aged from birth to 12 years. This age profile was selected as children older than 12 years are less likely to require liquid medicines than children 12 years and under. In Ireland, a pharmacist can switch brand of a medicine dispensed unless the prescriber specified otherwise, and can change the strength of a formulation dispensed, but they cannot change the type of antibiotic dispensed without contacting the prescriber.

### Sample characteristics

The inclusion criteria were GPs and trainee GPs registered with the Medical Council of Ireland, and pharmacists registered with the Pharmaceutical Society of Ireland, who selected community pharmacy as their area of professional practice.

### Data collection methods

Permission was granted by the Pharmaceutical Society of Ireland to use their community pharmacist email contact list. The questionnaire was emailed to 3,943 community pharmacists inviting them to take part in the study. The research team did not have access to a national database of GP email addresses. Therefore, the questionnaire was disseminated to GPs and trainee GPs in the Cork region of Ireland using the Cork GP training scheme email list which includes 64 GPs and GP trainees. A link to the online questionnaire was included in the email. To maximise study reach and participation, a link to the questionnaire was posted by members of the study team and related institutional accounts on Twitter and LinkedIn and via relevant professional discussion groups on WhatsApp. The questionnaire was available from November 2021 to March 2022.

### Data analysis

The responses from the online questionnaire were analysed in Microsoft Excel. Categorical variables were presented as frequencies (%) and responses to free-text questions were explored using thematic analysis. As each survey item was not compulsory, percentage responses were calculated based on the number of responses to that individual survey item.

## Results

### Participant demographics

A total of 244 participants completed the questionnaire and key demographic details are outlined in Supplementary Table S1. Fifty-nine respondents were GPs (24.2%) and the remaining 185 participants were pharmacists (75.8%). As the survey was distributed via social networking sites, a final response rate could not be determined. One hundred and seventy-three of the participants were female (70.9%). Twenty-nine GPs (49.2%) and 134 pharmacists (72.4%) had >10 year’s professional experience. Thirty-seven GPs (64.9%) and 141 pharmacists (77.5%) reported that ≤20% of their prescription patients were children.

### General factors that influence the antibiotic prescribing and dispensing

When asked to rank factors they considered when prescribing or dispensing an oral liquid antibiotic product for children, clinical guidelines and availability of supply were ranked as the most important factors by the majority of GP and pharmacist respondents respectively (Table 1). While a minority of respondents ranked palatability as the most important factor, 38% of each profession listed it as the second important factor from the choices provided.

**Table 1.** Factors that influence prescribing of oral liquid antibiotic by GP and dispensing of oral liquid antibiotics by pharmacist respondents respectively.

Rank	GPs (*n* = 59)			Pharmacists (*n* = 185)		
	Clinical guidelines	Dosage regime	Palatability	Availability of supply	Product cost	Palatability
Most important factor	79.7% (*n* = 47)	6.8% (*n* = 4)	13.6% (*n* = 8)	66.5% (*n* = 123)	13.5% (*n* = 25)	20% (*n* = 37)
Second important factor	16.9% (*n* = 10)	45.8% (*n* = 27)	37.3% (*n* = 22)	16.2% (*n* = 30)	45.9% (*n* = 85)	37.8% (*n* = 70)
Least important factor	3.4% (*n* = 2)	47.5% (*n* = 28)	49.2% (*n* = 29)	17.3% (*n* = 32)	40.5% (*n* = 75)	42.2% (*n* = 78)

Participants then listed any other factors they consider when prescribing or dispensing an oral liquid antibiotic product for children. The question was answered by 76 pharmacists (41.1%) and 20 GPs (33.9%) and a wide range of factors was noted. The 144 factors mentioned were categorised into factors associated with patient and product. While patient factors were listed by both GPs and pharmacists, they were reported more predominantly by GPs. A number of responses were similar across both professions, including allergy, age appropriateness, antimicrobial history, customer preference, and patient feedback. However, other patient factors reported by GPs and not by pharmacists included parental attitude and education, child’s weight, compliance and illness. In contrast, product factors were more frequently listed by pharmacists. Product factors noted by both professions included duration of therapy and route of administration. However, dose volume was the main product factor identified by 21.3% of pharmacists. More details are outlined in Supplementary Table S2.

### Palatability of oral antibiotic type

**Fig. 1.**
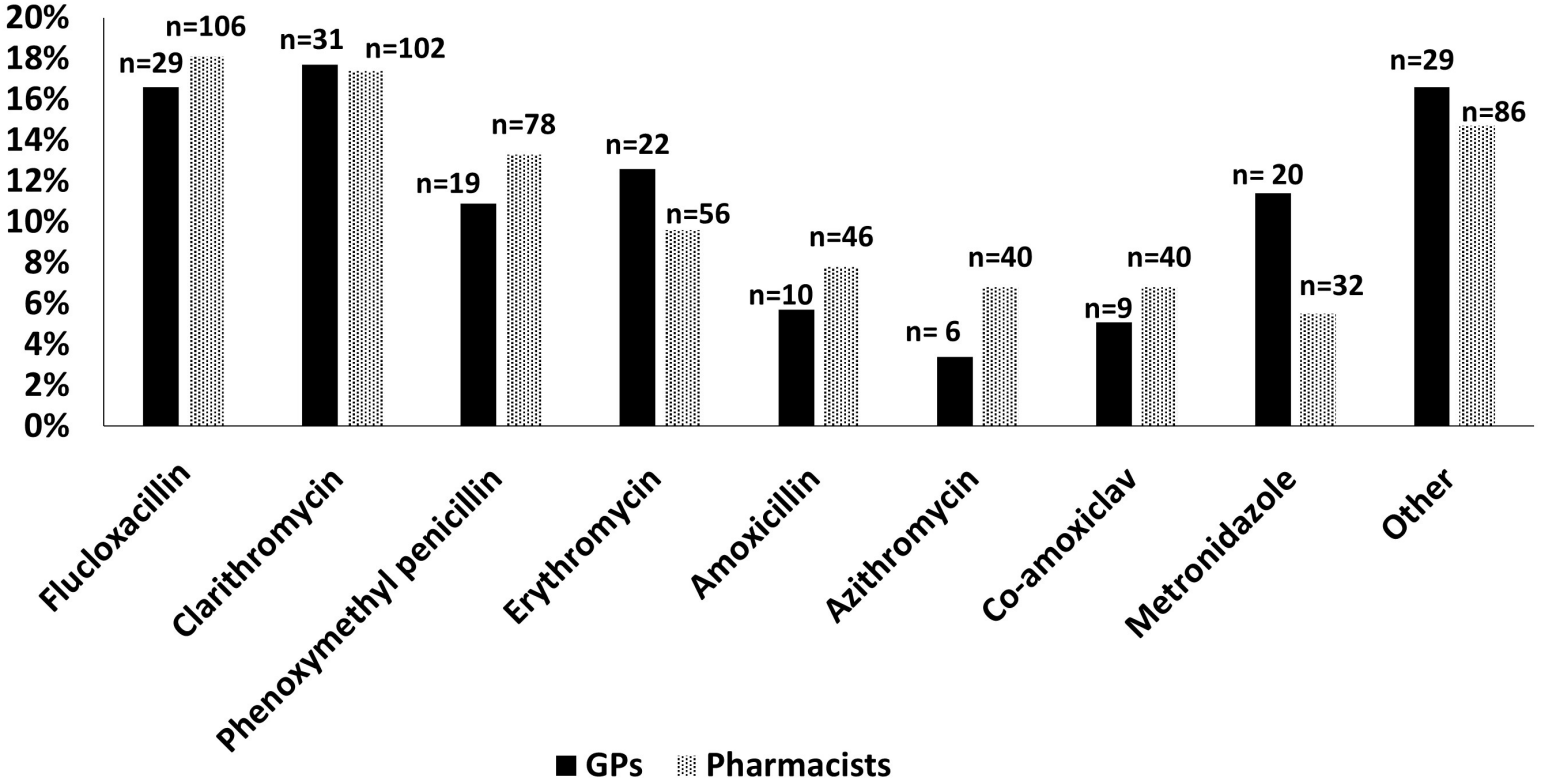
Percentages of antibiotics with palatability issues (e.g. smell, taste, aftertaste, and texture) reported by the survey participants. A total of 175 responses from GPs and 587 responses from pharmacists were received for this question.

Flucloxacillin and clarithromycin were identified as the most unpalatable antibiotics by both professions (Fig. 1).

**Fig. 2.**
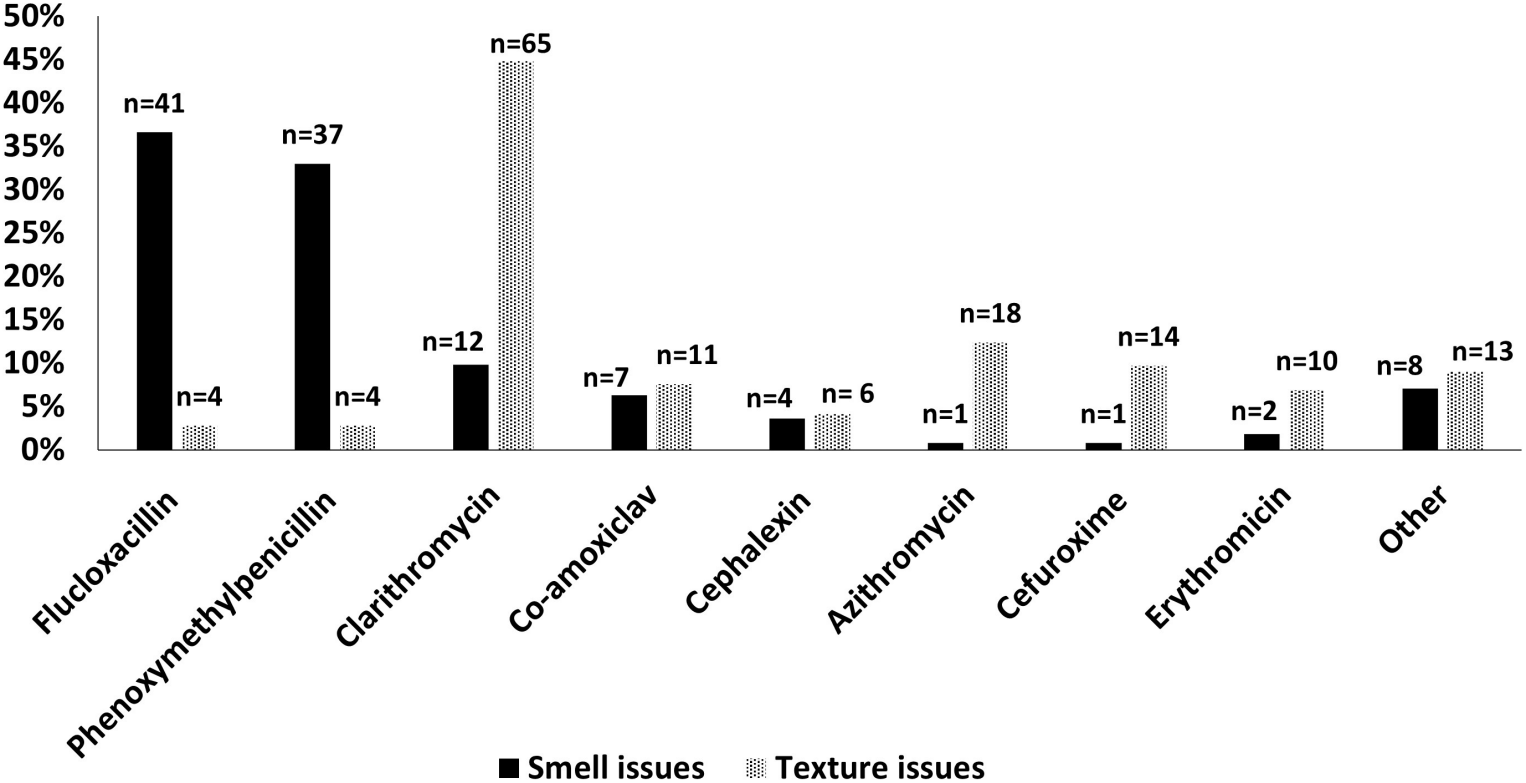
Percentages of antibiotics with smell and texture issues (i.e. feeling in the mouth) that pharmacist respondents had encountered during their preparation. One hundred and twelve responses were received for the smell issue and 145 responses were received for the texture issue.

When pharmacists were asked to state oral liquid antibiotic products with smell issues, one hundred and twelve responses were received. The antibiotics reported with the worst smell were flucloxacillin (*n* = 41, 36.6%) and phenoxymethylpenicillin (*n* = 37, 33.0%). When the pharmacists were then asked to state oral liquid antibiotic products with texture issues, one hundred and forty-five responses were received from 135 respondents. Clarithromycin was most frequently identified as an antibiotic with texture issues (*n* = 65, 44.8%). Azithromycin (*n* = 18, 12.4%) and cefuroxime (*n* = 14, 9.7%) were also reported to have texture issues (Fig. 2).

Amoxicillin was reported as the most palatable antibiotic for children by 58.1% of GP respondents and 49.7% of pharmacist respondents. Cefaclor was identified as the most palatable oral liquid antibiotic by 19.4% of GP respondents and 25.1% of pharmacist respondents.

### Palatability impact on clinical practice

**Fig. 3.**
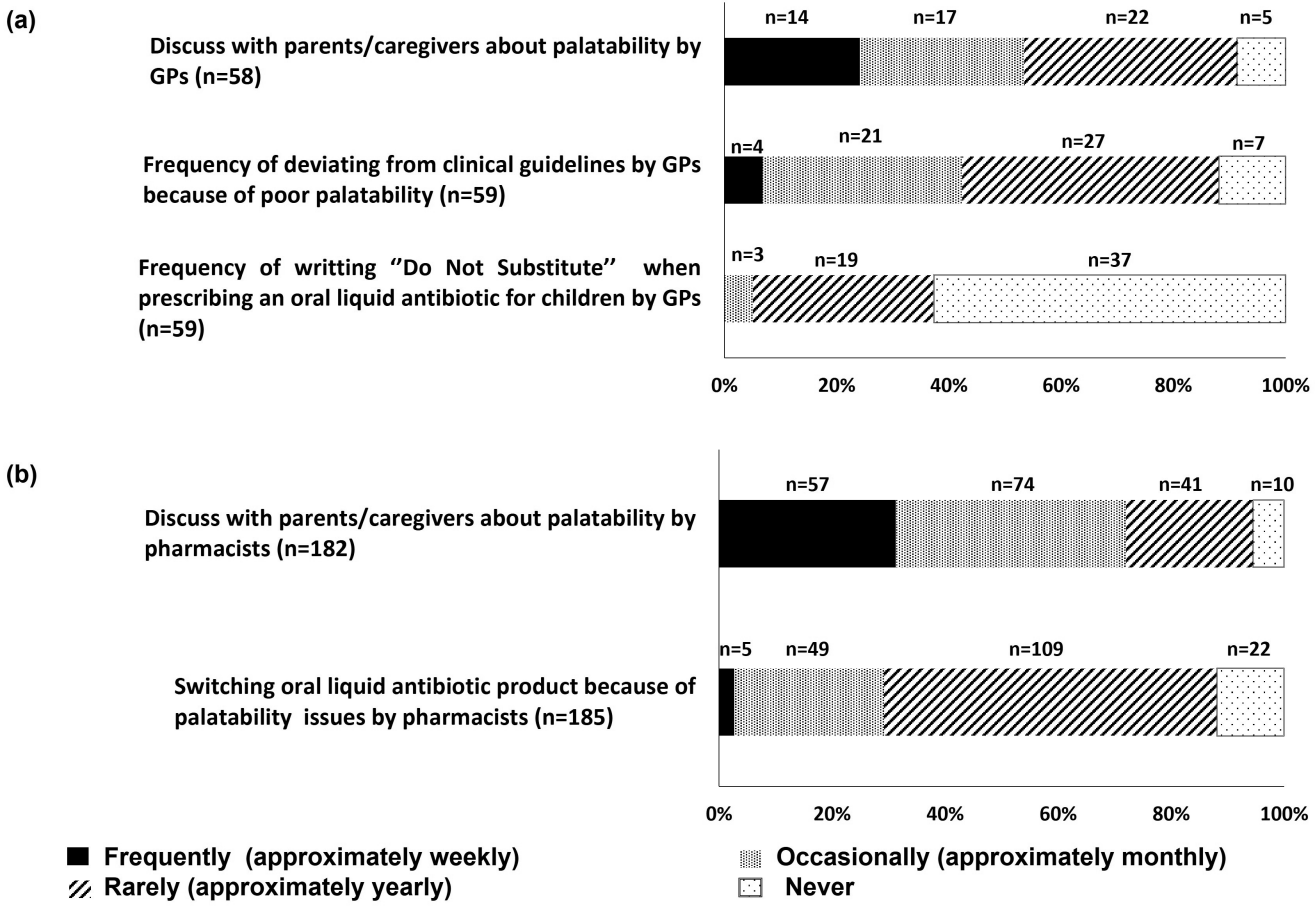
Discussion about palatability (e.g. smell, taste, aftertaste, and texture) with parents/caregivers and frequency of deviating from clinical guidelines and switching oral liquid antibiotics for children reported by a) GPs and b) pharmacists’ respondents respectively.

Of the pharmacists surveyed, 31.3% frequently and 40.7% occasionally discussed antibiotic palatability with parents/caregivers. However, 46.6 % of GPs indicated that they rarely or never discussed the palatability with parents/caregivers (Fig. 3). Among GP respondents (*n* = 59), 62.7% reported never and 32.2% reported rarely writing “Do not substitute” on a prescription for an oral liquid antibiotic for a child (Fig. 3). Among both GPs and pharmacists, 11.9% indicated that they never deviated from clinical guideline or changed dispensing choice because of palatability issues respectively. However, occasional (approximately monthly) deviation from clinical guidelines was reported by 35.6% GPs, and occasional switching was reported by 26.5% of pharmacists (Fig. 3).

GP respondents who had deviated from clinical guidelines were asked to rank the palatability-related reasons causing them to deviate from clinical guidelines (Table 2). Of the 52 respondents, 40 (76.9%) reported that ensuring adherence to treatment was the most common reason to deviate from clinical guidelines. Maintaining a good physician–patient relationship was ranked as the second common reason by 50% of GP respondents. GP participants were then asked a free text question to list any other reasons that cause them to deviate from clinical guidelines because of poor palatability. One respondent stated that oral liquid antibiotics dosed four times a day are very awkward for school-going children.

**Table 2.** Reasons which cause GPs to deviate from clinical guidelines because of poor palatability (*n* = 52).

Rank	Ensure compliance and adherence to treatment	Maintain a good physician–patient relationship	Parental/caregiver pressure
The most common reason	76.90% (*n* = 40)	7.70% (*n* = 4)	15.40% (*n* = 8)
The second common reason	13.50% (*n* = 7)	50.00% (*n* = 26)	36.50% (*n* = 19)
The least common reason	9.60% (*n* = 5)	42.30% (*n* = 22)	48.10% (*n* = 25)

### Switching antibiotic products by pharmacists because of palatability issues

Pharmacists who reported that they had switched to an oral liquid antibiotic product because of palatability issues were asked to indicate which products they had switched from and to. The question was answered by 113 pharmacists with 143 individual responses. Switching from one brand to another brand of the same antibiotic was reported by 30.8% of respondents. This included switching brands of phenoxymethylpenicillin (*n* = 24), flucloxacillin (*n* = 7), amoxicillin (*n* = 6), and other (*n* = 7). A further 44.8% of the respondents reported switching from an antibiotic to a different antibiotic after consulting the prescriber. Respondents reported contacting the prescriber for alternative prescriptions after parents had complained of non-compliance. Most of the participants reported that they switched from clarithromycin (*n* = 17), flucloxacillin (*n* = 13), and phenoxymethylpenicillin (*n* = 12). These antibiotics were switched mainly to either amoxicillin (*n* = 12) or cefaclor (*n* = 11). Twenty-six participants identified which antibiotic they had switched from without specifying which product they had switched to. Examples of switching are outlined in Supplementary Table S3 and S4.

### Advice to administer an unpalatable oral liquid antibiotic to a child

Pharmacists were asked how they would identify an oral liquid antibiotic product that may have poor palatability. Of the total responses (*n* = 379), 47% were identified through parent/caregiver feedback. Palatability issues were also identified through drug smell during preparation (20.6%), drug texture during preparation (16.4%), parents/caregiver unwilling to administer the drug to the children (14.0%), and 2.1% stated their own personal experience.

Pharmacists were then asked about the advice they would give to a parent/caregiver who is unable to administer an unpalatable oral liquid antibiotic to a child. Of the 289 responses, 52% reported advising a parent/caregiver to manipulate the required doses, such as by mixing with food or diluting the antibiotic in a flavoured liquid; 21.8% advised to promise a reward to the child after taking the dose; and 14.2% recommended dividing the required dose into smaller portions. Other advice reported by pharmacists is outlined in Supplementary Table S5.

## Discussion

This study has identified palatability issues associated with prescribing and dispensing oral liquid antibiotics for paediatric patients reported by GPs and pharmacists. Among GPs and pharmacists, 88.1% of both professions reported that they have deviated from clinical guidelines or changed dispensing choice because of palatability issues respectively. Among GPs who have deviated from clinical guidelines, 77% reported this was in order to ensure compliance and adherence to treatment. Among pharmacists who have changed dispensing choice due to palatability issues, 31% report that they switch from one brand to a more palatable brand of the same antibiotic, and 45% switch the type of the antibiotic after consulting the GP. In some cases, where a parent/caregiver is unable to administer an unpalatable oral liquid antibiotic to a child, pharmacists are advised to manipulate the required doses by mixing them with food or diluting them in a flavoured liquid. Flucloxacillin and clarithromycin were reported as the least palatable oral liquid antibiotics for children. Flucloxacillin was highlighted as the antibiotic with the worst smell whilst clarithromycin was the antibiotic with the most textural issues. Amoxicillin and cefaclor were identified as the most palatable oral liquid antibiotics for a paediatric population.

In the current study, 80% of GPs identified clinical guidelines as the most important factor they considered when prescribing an oral liquid antibiotic for children. However, 88% of them reported that they might need to deviate from clinical guidelines because of poor palatability, an issue that was also reported by Bergene et al. who found that GPs preferred to follow the guidelines and prescribe first-choice antibiotics. However, poor palatability could lead to prescribing of second-choice agents.^15^ Additionally, previous studies showed that poor palatability is a main reason for prescribing second-choice, broad-spectrum antibiotics for paediatric patients.^27–29^ In the present study, GPs reported that their primary reason for deviating from clinical guidelines was to ensure adherence to treatment. The second most common reason was to maintain a good physician–patient relationship. It has previously been reported that sub-optimal adherence to treatment might cause complications in the physician–patient relationship.^30^

Among pharmacists in the present study, 67% identified the availability of supply as the most important factor they considered when dispensing an oral liquid antibiotic for children. However, 88% reported that they would switch antibiotic products because of poor palatability. Pharmacists reported switching from a less palatable brand to a more palatable brand of the same antibiotic or switching the type of the antibiotic, based on parent/caregiver feedback. This was done after consulting with the prescribing GP. These results are similar to those reported by Bergene et al., who found that pharmacists might need to switch the prescribed antibiotic product at parents’ request or change the type of antibiotic after contacting the prescriber.^15^ Pharmacists in the current study reported that they had contacted the prescriber for alternative prescriptions after parents had complained of non-adherence. Pharmacists revealed that where a parent/caregiver is unable to administer an unpalatable oral liquid antibiotic to a child, the pharmacist might advise to manipulate the required doses or promise a reward to the child after taking the dose. However, Venables et al. have reported that unnecessary manipulation should be avoided as such manipulation can alter therapeutic response and decrease drug bioavailability.^20^ Similarly, a previous study reported that parents/caregiver might need to manipulate the oral dosage form to achieve better palatability.^31^ The study reported the need to develop more palatable formulations in order to reduce drug manipulation.

Palatability is discussed with parents/caregivers by 91% of GPs and 95% of pharmacists when prescribing or dispensing oral liquid antibiotics for children. Such discussion is essential to ensure successful treatment.^7^ An effective collaboration between GP, pharmacist, parent, and child can enhance the appropriate selection of antibiotic, improve adherence to treatment and hence decrease the risk of antimicrobial resistance.^7,15^ Baguley et al. suggest that GPs need to be more aware of the importance of palatability when prescribing oral liquid antibiotics for children to ensure effective prescribing.^7^ Bergene et al. recommend that pharmacists could integrate shared decision-making into their practice and that they could discuss the availability of different formulations with a parent/caregiver in order to dispense the best choice oral liquid antibiotic for every child.^15^ The three strategies of drug administration of an oral liquid antibiotic are open administration, hidden administration, and forced administration.^19^ It is essential that both GPs and pharmacists discuss these three strategies with the parent/caregiver and identify their preferences when prescribing or dispensing an oral liquid antibiotic.^19^

The most palatable oral liquid antibiotics for children were reported in the current study to be amoxicillin and cefaclor. However, cefaclor a cephalosporin broad-spectrum antibiotic, should not be used as a first-line choice when a narrow-spectrum antibiotic is effective in primary care, according to the current clinical guidelines in Ireland and the United Kingdom.^32,33^ Nevertheless, 18% of pharmacists in the present study highlighted that cefaclor was one of the antibiotics that they had frequently switched to after contacting the GP because of its preferred palatability.

In the current study, flucloxacillin and clarithromycin were reported to be the least palatable oral liquid antibiotics for children. A number of previous studies have reported the unpalatability of clarithromycin to paediatric patients.^6,34–36^ Steele et al. reported that the texture and acceptability of clarithromycin can be improved by decreasing the grittiness of the product in pharmaceutical processing.^4^ However, the findings of the current study suggest that the texture of clarithromycin is still problematic to children. Flucloxacillin has been identified in the past as an unpalatable oral liquid antibiotic for children.^7,21,37^ However, unlike Venables et al., who reported that the unpalatability of flucloxacillin relates to its taste,^21^ the findings of the present study indicate that the poor palatability of flucloxacillin relates to its smell. Smell is considered to be the main sense that contributes to flavour.^38,39^ It was confirmed in a review conducted by Spence that between 75% and 95% of what we think of as taste is actually caused by the smell sense.^40^ Indeed, olfaction was agreed by most researchers in the review by Spence to be the dominant sense in tasting.^40^ However, there is ongoing confusion with using the term “taste” to express the sense of flavour.

These findings highlight the importance of developing more palatable oral liquid formulations of the first-line antibiotics for paediatric population to avoid using broad-spectrum antibiotics and to ensure better adherence to the clinical guidelines. Availability of such improved formulations has the potential to improve patient adherence, reduce antimicrobial treatment failure, and improve the care of paediatric patients.

## Strengths and limitations

This study included both GPs and pharmacists to provide a comprehensive explanation of the impact of drug palatability on prescribing and dispensing processes of oral liquid antibiotics. Additionally, the respondents are very experienced with at least half in each profession having >10 years’ experience in their discipline. However, there are limitations to this study. The number of GP respondents was relatively small as the survey was disseminated by email to GPs and trainee GPs in the Cork region only. The survey was distributed in November 2021, during the Covid-19 pandemic, when all healthcare services including GPs and pharmacists, were under pressure. This may account for the small sample size. The survey was also circulated on social media to ensure a broader recruitment of eligible participants. There is a risk that in circulating the survey on social media, it could reach people who are not eligible to participate in the study. However, this topic is very specific and needed someone in clinical practice to answer the questions. To moderate this risk, the public social media posts specified that Irish-based participants were sought for the study and specified the two professions targeted. The WhatsApp messages were sent to groups that were specifically for Irish-based GPs and pharmacists. The relatively small sample size may limit the generalisability of the study. The survey instrument used in the study was novel and has not been used in any population outside of this study. However, the results align with those of qualitative studies that have been conducted in Norway, the United Kingdom, and the United States.

## Conclusion

This study has identified palatability issues associated with prescribing and dispensing oral liquid antibiotics for paediatric patients reported by GPs and pharmacists. A majority of GP respondents reported that they have deviated from clinical guidelines because of palatability issues. The need to ensure adherence to treatment was the main palatability-related reason causing GPs to deviate from clinical guidelines. Similarly, most pharmacists reported that they have switched an antibiotic due to palatability issues reported by a parent/caregiver. Pharmacists reported switching from one brand to a more palatable brand of the same antibiotic based on parents/caregiver feedback or switching the type of the antibiotic after consulting the GP. In some cases, where a parent/caregiver is unable to administer an unpalatable oral liquid antibiotic to a child, pharmacists advised to manipulate the required doses by mixing with food or diluting in a flavoured liquid. Amoxicillin and cefaclor were identified as the most palatable oral liquid antibiotics while flucloxacillin and clarithromycin were reported as antibiotics with poor palatability. There is a need to develop pharmaceutical approaches to adapt oral liquid formulations of flucloxacillin and clarithromycin antibiotics to improve palatability and thus paediatric patient acceptability.

## Supplementary material

Supplementary material is available at *Family Practice* online.

cmad071_suppl_Supplementary_Material

## Data Availability

Data are available and can be furnished upon reasonable request to the corresponding author.
