# Supplementary material for: The impact of drug palatability on prescribing and dispensing of antibiotic formulations for paediatric patients: a cross-sectional survey of general practitioners and pharmacists
Source: Fam Pract. 2023 Jul 6;41(6):962–9. doi: 10.1093/fampra/cmad071 (PMC11636568; doi:10.1093/fampra/cmad071)
Supplement: cmad071_suppl_Supplementary_Material [file cmad071_suppl_supplementary_material.pdf]

Supplementary Materials: The impact of drug palatability on prescribing and dispensing of antibiotic formulations for paediatric patients: a cross-sectional survey of general practitioners and pharmacists.

Survey Questionnaire

Title page: Attitudes and experiences of healthcare providers to prescribing and dispensing of oral liquid antibiotics for paediatric patients.

If you agree to take part in this study, please complete the consent form overleaf.

Consent Form

Do you consent to participate in this study? *

Yes ☐

No ☐

Survey questions

*Required

Section 1: General Section

1. What is your gender?

Please tick one box.

☐ Male

☐ Female

☐ Prefer not to say

☐ Other: ______

2. What is your clinical discipline? *

Please tick one box.

☐GP Skip to section 2

☐Pharmacist Skip to section 7

Section 2: GP profile section

3. What is your current GP status?

Please tick one box.

- ☐ GP principal
- ☐ GP non-principal
- ☐ Sessional GP
- ☐ Locum GP
- ☐ GP trainee
- ☐ Other:

4. How long have you worked in general practice?
Please tick one box.

- ☐ 0–4 years
- ☐ 5–10 years
- ☐ more than 10 years

Section 3: GP practice section

5. How many GPs are in your practice?
Please tick one box.

- ☐ Single handed
- ☐ 2-3
- ☐ 4–5
- ☐ more than 6

6. How would you characterise your practice location?
Please tick one box.

- ☐ Urban
- ☐ Rural
- ☐ Mixed urban and rural

7. What approximate percentage of your patients have a GMS full medical card? (your best estimate)

8. What approximate percentage of your patients are children (age of birth to 12 years)? (your best estimate)

Section 4: factors that influence the prescribing of oral liquid antibiotics.

9. Please rank the following factors you consider when prescribing an oral liquid antibiotic product for children (age of birth to 12 years): Rank 1 for the factor considered most important, 2 for next most important etc.*

☐ Dosage regime (e.g., once daily, twice daily, three times daily)

☐ Clinical guidelines

☐ Palatability (e.g., smell, taste, aftertaste, and texture)

10. If you have other factors you consider when prescribing an oral liquid antibiotic product for children, please list them.

11. How often in the past have you changed your oral liquid antibiotic prescription choice because of dosage regimen? (your best estimate)
Please tick one box.

☐ Frequently (approximately weekly)

☐ Occasionally (approximately monthly)

☐ Rarely (approximately yearly)

☐ Never

12. How often in the past have you deviated from clinical guidelines related to oral liquid antibiotic prescribing because of poor palatability? (your best estimate) *
Please tick one box.

☐ Frequently (approximately weekly) skip to section 5

☐ Occasionally (approximately monthly) skip to section 5

☐ Rarely (approximately yearly) skip to section 5

☐ Never skip to section 6

Section 5: Palatability of oral liquid antibiotics

13. Please rank the reasons which cause you to deviate from clinical guidelines because of medicine's poor palatability (e.g., smell, taste, aftertaste, and texture) (where 1 is the most common reason and 3 is the least common reason): *

☐ Ensure compliance and adherence to treatment

☐ Maintain a good physician-patient relationship

☐ Parental/ caregiver pressure

14. If you have other reasons which cause you to deviate from clinical guidelines because of medicine's poor palatability, please list them.

15. How often in the past have you written ‘’*Do Not Substitute’’* when prescribing an oral liquid antibiotic for children (age of birth to 12 years) because of palatability issues (e.g., smell, taste, aftertaste, and texture)? (Your best estimate)

Please tick one box.

☐ Frequently (approximately weekly)

☐ Occasionally (approximately monthly)

☐ Rarely (approximately yearly)

☐ Never

16. How often in the past have you discussed the palatability (e.g., smell, taste, aftertaste, and texture) of oral liquid antibiotics with parents/caregivers when prescribing? (Your best estimate)

Please tick one box.

☐ Frequently (approximately weekly)

☐ Occasionally (approximately monthly)

☐ Rarely (approximately yearly)

☐ Never

17. From the following list of oral liquid antibiotics, please select the medicines you are aware patients can experience palatability (e.g. smell, taste, aftertaste and texture) issues with: *

Tick all that apply.

☐ Amoxicillin

☐ Azithromycin

☐ Cefalexin

☐ Ciprofloxacin

☐ Clarithromycin

☐ Co-amoxiclav

☐ Co-trimoxazole

☐ Erythromycin

☐ Flucloxacillin

☐ Metronidazole

☐ Nitrofurantoin

☐ Phenoxymethylpenicillin

☐ Trimethoprim

☐ Other:

18. Which oral liquid antibiotic do you think is the most palatable to children (age of birth to 12 years)? Indicate specific brand if possible. *

19. Which oral liquid antibiotic do you think is the least palatable to children (age of birth to 12 years)? Indicate specific brand if possible. *

20. We would like to further explore the issues around the palatability of oral liquid antibiotics for children. If you are interested in taking part in a telephone/online interview (approximately 15 minutes) on this research topic, please provide your contact details:

Name:

Phone number:

Email address:

Section 6: Palatability of oral liquid antibiotics

Similar to Section 5 (Questions 15-20) with question 13,14 excluded

Section 7: Pharmacist profile section

3. Which title would describe your current practice of pharmacy?
Please tick one box.

- ☐ Supervising community pharmacist
- ☐ Non supervising community pharmacist
- ☐ Locum community pharmacist
- ☐ Other:

4. How long have you been working as a community pharmacist?
Please tick one box

- ☐ 0–4 years
- ☐ 5–10 years
- ☐ more than 10 years
- ☐ Other:

Section 8: Pharmacist practice section

5. How many full-time equivalent pharmacists work in your community pharmacy?
Please tick one box.

☐ 1-2 pharmacists

☐ 3-5 pharmacists

☐ More than 5

6. What type of pharmacy business do you work in?
Please tick one box.

☐ Large multiple of eleven or more pharmacies

☐ Medium chain of 6 to 10 pharmacies

☐ Small chain of 5 to 2 pharmacies

☐ Single independent pharmacy

7. How would you characterise the community pharmacy you work in?
Please tick one box.

☐ Urban

☐ Rural

☐ Mixed urban and rural

8. What approximate percentage of your prescription patients have a GMS full medical card? (Your best estimate)

9. What approximate percentage of your prescription patients are children (age birth to 12 years)? (Your best estimate)

Section 9: Factors that influence the dispensing of oral liquid antibiotics.

10. Please rank the following factors you consider when dispensing an oral liquid antibiotic product for children (where 1 is the most important factor and 3 is the least important factor) *

☐ Availability of supply

☐ Product cost

☐ Palatability (e.g. smell, taste, aftertaste and texture)

11. If you have other factors you consider when dispensing an oral liquid antibiotic product for children, please list them.

12. How often in the past have you switched an oral liquid antibiotic product because of dosage regimen? (your best estimate)
Please tick one box.

☐ Frequently (approximately weekly)

☐ Occasionally (approximately monthly)

☐ Rarely (approximately yearly)

☐ Never

13. How often in the past have you switched an oral liquid antibiotic product because of palatability issues (e.g., smell, taste, aftertaste and texture)? (your best estimate) *
Please tick one box.

☐ Frequently (approximately weekly)	skip to section 10
☐ Occasionally (approximately monthly)	skip to section 10
☐ Rarely (approximately yearly)	skip to section 10
☐ Never	skip to section 11

Section 10: Palatability of oral liquid antibiotics (1)

13.a. If you have switched an oral liquid antibiotic product/s because of palatability issues (e.g. smell, taste, aftertaste and texture), please indicate which products you have switched from and which products you have switched to?

14. If you have switched an oral liquid antibiotic product/s because of dosage regimen, please indicate which products you have switched from and which products you have switched to?

15. From the following list of oral liquid antibiotics, please select the medicines you are aware patients can experience palatable issues with: *
Tick all that apply.

☐ Amoxicillin

☐ Azithromycin

☐ Cefalexin

☐ Ciprofloxacin

☐ Clarithromycin

☐ Co-amoxiclav

☐ Co-trimoxazole

☐ Erythromycin

☐ Flucloxacillin

☐ Metronidazole

☐ Nitrofurantoin

☐ Phenoxymethylpenicillin

☐ Trimethoprim

☐ Other: ______

16. Which oral liquid antibiotic do you think is the most palatable to children (age from birth to 12 years)? Indicate specific brand if possible. *

______

17. Which oral liquid antibiotic do you think is the least palatable to children (age from birth to 12 years)? Indicate specific brand if possible. *

______

18. How would you identify an oral liquid antibiotic product that may have poor palatability?

Tick all that apply.

☐ Parent/caregiver feedback

☐ Parent/caregiver unwilling to administer the drug to the children

☐ Drug smell during preparation

☐ Drug texture during preparation

☐ Other: ______

Section 11: Palatability of oral liquid antibiotics (1)

Similar to Section 10 (Questions 14-18) with question 13.a excluded.

Section 12: Palatability of oral liquid antibiotics (2)

19. What oral liquid antibiotic product/s with smell issues have you encountered during their preparation? State the brand name of such product(s) if applicable.

20. What oral liquid antibiotic product/s with texture issues have you encountered during their preparation? State the brand name of such product(s) if applicable.

21. How often do you discuss with parents/caregivers about oral liquid antibiotic palatability when dispensing? (your best estimate)

Please tick one box.

☐ Frequently (approximately weekly)

☐ Occasionally (approximately monthly)

☐ Rarely (approximately yearly)

☐ Never

22. What advice would you give to a parent/ caregiver who is unable to administer an unpalatable oral liquid antibiotic to children?

☐ Dividing the required dose into smaller portions

☐ Manipulate the required doses (e.g., mixing with food or diluting in a flavoured liquid)

☐ Promising a reward to the child after taking dose

☐ Other:

23. We would like to further explore the issues around the palatability of oral liquid antibiotics for children. If you are interested in taking part in a telephone/online interview (approximately 15 minutes) on this research topic, please provide your contact details:

Name:

Phone number:

Email address:

Table S1. Healthcare professional demographics. Total responses n = 244.

Parameters	GPs % (n)	Pharmacists % (n)
**Clinical discipline**	24.2 (59)	75.8 (185)
**Gender**<br>Male<br>Female<br>Prefer not to say	 32.2 (19)<br>66.1 (39)<br>1.7 (1)	 26.5 (49)<br>72.4 (134)<br>1.1 (2)
**Role**	GP principals 7.5 (28)<br><br>GP trainees 35.6 (21)<br><br>GP non-principals 8.5 (5)<br><br>Sessional GPs 6.8 (4)<br><br>Other 1.7 (1)	Supervising community pharmacist* 51.1 (94)<br><br>Non supervising community pharmacist 33.2 (61)<br><br>Locum community pharmacist** 13.6 (25)<br><br>Other 2.2 (4) & **Missing (n= 1)**
**Period of working**<br>0–4 years<br>5–10 years<br>more than 10 years	 37.3 (22)<br>13.6 (8)<br>49.2 (29)	 14.1 (26)<br>13.5 (25)<br>72.4 (n=134)
**Full-time staff working in the practice**	Single handed 8.5 (5)<br><br>2-3 GPs 39 (23)<br><br>4–5 GPs 37.3 (22)<br><br>More than 6 GPs 15.3 (9)	1-2 pharmacists 76.1 (140)<br><br>3-5 pharmacists 21.7 (40)<br><br>More than 5 pharmacists 2.2 (4)<br><br>**Missing (n= 1)**
**Practice location**<br>Urban<br>Rural<br>Mixed urban and rural	 44.8 (26)<br>27.1 (16)<br>27.1 (16) & **Missing (n= 1)**	 43.8 (81)<br>19.5 (36)<br>36.8 (68)
**Type of pharmacy business**<br>Large multiple of 11 or more<br>Medium chain of 6 to 10<br>Small chain of 5 to 2<br>Single independent pharmacy		 24.5 (45)<br>8.7 (16)<br>23.4 (43)<br>43.5 (80) & **Missing (n= 1)**
**Percentage of prescription patients with a GMS full medical card**<br>0-50%<br>51-100%	 36.8 (21)<br>63.2 (36) & **Missing (n=2)**	 36.8 (68)<br>63.2 (117)
**Percentage of prescription patients are children**<br>0-20%	 64.9 (37)	 77.5 (141)

* Supervising community pharmacist is the person responsible for the day-to-day management and operation to the pharmacy.

** Locum community pharmacist is a pharmacist employed on a contractual basis through an agency, rather than having a permanent salaried position.

20-50%<br>51-100%	35.1 (20)<br>0 (0) & **Missing (n=2)**	20.9 (38)<br>1.6 (3) & **Missing (n=3)**

Table S2. Other factors that influence prescribing and dispensing of oral liquid antibiotics reported by GP and pharmacist respondents. Responses were received from 76 pharmacists with 122 responses and from 20 GPs with 20 responses.

Other factors	List of the factors	GPs % (n)	Pharmacists % (n)
**Patient factors**	Allergy	20 (4)	6.6 (8)
	Customer preference, patient feedback	10(2)	3.3 (4)
	Antimicrobial history	10 (2)	0.8 (1)
	Age appropriate	5 (1)	4.1 (5)
	Ability to swallow or whether the child can take tablet medication	5 (1)	0.8 (1)
	Parental attitude and education	15 (3)	-
	Child's weight	10 (2)	-
	Compliance	5 (1)	-
	Illness	5 (1)	-
	Co-morbidities	-	3.3 (4)
	Ease of dosing delivery	-	2.5 (3)
**Product factors**	Duration of therapy	5 (1)	4.1 (5)
	Route of administration	5 (1)	1.6 (2)
	Cost	5 (1)	-
	Dose volume	-	21.3 (26)
	Frequency	-	2.5 (3)
	Shelf life	-	7.4 (9)
	Stability	-	2.5 (3)
	Storage	-	6.6 (8)
	Strength	-	4.9 (6)
	What is prescribed	-	4.9 (6)
	Indication	-	4.1 (5)
	Preferred generic	-	3.3 (4)
	Immediate use or dry, easy to disperse when given dry	-	3.3 (4)
	Expiry date	-	2.5 (3)
	Ease of measuring dose	-	2.5 (3)
	Choice of manufacturer	-	2.5 (3)
	Pack size	-	1.6 (2)
	Discount	-	0.8 (1)
	Dosage form	-	0.8 (1)
	appropriate labelling	-	0.8 (1)
	Viscosity	-	0.8 (1)

Table S3. Switching antibiotic products by pharmacists because of palatability issues. 113 pharmacists answered this question with 143 responds of which 9 were could not remember.

Antibiotics	Examples revealed of switching between products of the same antibiotic (n=44)	Examples revealed of switching to a different antibiotic (n=64)	Examples revealed of products switched from without specifying which product switched to (n=26)

Phenoxymethylpenicillin (33.6%, n=38)	Kopen® to Calvepen® (n=17) and from Calvepen® to Kopen® (n=7)	Calvepen® to Amoxicillin® (n=4), penicillin v to Cefaclor® (n=1), penicillin v to Oramox® (n=1). Calvepen® to another antibiotic (n=6)	Calvepen® (n=2)
Clarithromycin (25.7%, n=29)	-	Klacid® to Distaclor® (n=10), Klacid® to another antibiotic (n=7)	Klacid® (n=12)
Flucloxacillin (22.1%, n=25)	Switching to Floxapen® (n=3), from liquid to tablet (n=1), between flucloxacillin brands (n=3)	Flucloxacillin® to Amoxicillin® (n=7), Flucloxacillin® to another antibiotic (n=6)	Flucloxacillin® (n=5)
Amoxicillin (11.5%, n=13)	Oramox® to Amoxicillin® (n=1) and Clonamox® to Amoxicillin® (n=1), between amoxicillin brands (n=4)	Pinamox® to Augmentin duo® (n=2), Amoxicillin® to Distaclor® (n=2), Amoxicillin® to another antibiotic (n=3)	-
Co-amoxiclav (10.6%, n=12)	Germentin® to Augmentin® (n=1)	Augmentin® to Distaclor® (n=2), Augmentin® to Amoxicillin® (n=3), Augmentin® to another antibiotic (n=3)	Augmentin duo® (n=2), Augmentin® (n=1)
Other (15%, n=17)	From a flavour to another flavour (n=5), from Primacine® to Erythroped® (n=1)	Zinnat® to Distaclor® (n=2), Erythroped® to Distaclor® (n=1), Kefflex® to Amoxicillin® (n=1) or to Augmentin duo® (n=1), Zithromax® to Erythrocin® (n=1), from macrolide to penicillin (n=1)	Zithromax®, Erythromycin®, Suprax®, and Furantoin®

Switching antibiotics because of dosage regimen

All respondents were asked how often in the past had they switched an oral liquid antibiotic product for children because of dosage regimen. Almost half of the pharmacists (47%) and GPs (51%) indicated that they rarely (approximately yearly) changed dispensing or prescription choice because of dosage regimen. Similarly, occasionally (approximately monthly) was reported by 37.3% and 39%. of pharmacists and GPs respectively. It was apparent that the same proportion of both professions (10%) reported that they never switched for this reason.

Pharmacists who reported that they had switched an oral liquid antibiotic product because of dosage regimen (89.7%, n=166) were asked to indicate which products they had switched from and which products they had switched to. Eighty participants (48.2%) responded to this question of whom 14 were unsure or cannot recall.

28.8% of the participants switched from low strength (125mg/5ml) to high strength (250mg/5ml) either of the same product or between products of the same antibiotic (n=23). Changing to a more

concentrated products was reported by the participants that it reduces the volume for poor palatability, avoid needing to dispense a second bottle by halving the dosage volume, easier to disguise in food, and decrease the compliance issue. In contrast, 6.3% switched from high strength (250mg/5ml) to low strength (125mg/5ml) of the same product (n=5). However, 15% of the participants reported switching between co-amoxiclav products (n=12). Examples of the products reported are shown in ***Table S4***.

Table S4 Switching antibiotic products by pharmacists because of dosage regimen. 80 pharmacists responded to this question of whom 14 were unsure or cannot recall.

Switching antibiotic products by pharmacists because of dosage regimen	Examples of switching strengths of the same product	Examples of switching between products of the same antibiotic	Examples of switching to a different antibiotic
Switching from low strength (125mg/5ml) to high strength (250mg/5ml) of the same product (28.8%, n=23)	Pinamox® (n=4), Amoxicillin® (n=3), Oramox® (n=1), Kopen® (n=1)	From Kopen® to Calvapen® (n=3), and from Amoxicillin® generic to Amoxil® (n=1)	-
Switching from high strength (250mg/5ml) to low strength (125mg/5ml) of the same product (6.3%, n=5)	Amoxicillin® (n=2), Oramox® (n=1)	-	-
Switching between co-amoxiclav products (15%, n=12)	-	From Augmentin junior® and Augmentin paediatric® to Augmentin duo® (n=5), from Augmentin duo® to Augmentin® (n=2), and Germentin® to Augmentin® (n=1)	-
Other (16.3%, n=13)	Switching between various strengths such as Amoxicillin® (n=4), Flucloxacillin® (n=1)	Switching from solid to liquid dosage forms (n=5)	From Flucloxacillin® to Co-amoxiclav® (n=1), to Augmentin® (n=1), or to Augmentin duo® (n=1)

Table S5 Other Advice would pharmacists give to a parent/caregiver who is unable to administer an unpalatable oral liquid antibiotic to a child with a total of 37 responses from pharmacists for this question.

Advice would a pharmacist give to a parent/ caregiver	% (n)
Using a syringe aimed to back of the mouth to avoid tastebuds and administer small amounts into side of the mouth	32.4 (12)
Taking water or a flavoured drink afterwards to wash the medicine down	13.5 (5)
Switching to an alternative antibiotic if unable to take	8.1 (3)

Distraction techniques	8.1 (3)
Treat the child like a grown up and highlight the importance of the medicine	8.1 (3)
Mix with soft food such as chocolate syrup, yoghurt or squash	5.4 (2)
Suck an ice pop or ice cube beforehand to numb the tastebuds	5.4 (2)
If possible, use higher dose & less volume to administer	2.7 (1)
Ask how manageable the palatability issues are	2.7 (1)
Praising the child for taking the antibiotic	2.7 (1)
Offering the child, a reward	2.7 (1)
Ensure the full dose is consumed	2.7 (1)
If pain relief is due give the immediately after to mask taste	2.7 (1)
Changing to tablets for children 8+	2.7 (1)
